# Living with singleness: needs and concerns of never-married women over 35

**DOI:** 10.1186/s40359-021-00635-1

**Published:** 2021-08-30

**Authors:** Shakiba Pourasad Shahrak, Serge Brand, Ziba Taghizadeh

**Affiliations:** 1 Department of Midwifery and Reproductive Health, School of Nursing and Midwifery, Tehran University of Medical Sciences, Tehran, Iran; 2 Center for Affective, Stress and Sleep Disorders (ZASS), Psychiatric Clinics (UPK), University of Basel, 4002 Basel, Switzerland; 3 Division of Sport Science and Psychosocial Health, Department of Sport, Exercise, and Health, University of Basel, 4052 Basel, Switzerland; 4 Substance Abuse Prevention Research Center, Health Institute, Kermanshah University of Medical Sciences, Kermanshah, 67146 Iran; 5 Sleep Disorders Research Center, Kermanshah University of Medical Sciences, 67146 Kermanshah, Iran; 6 School of Medicine, Tehran University of Medical Sciences, 25529 Tehran, Iran; 7 Nursing and Midwifery Care Research Center, Department of Midwifery and Reproductive Health, Tehran University of Medical Sciences, Tehran, Iran

**Keywords:** Never-married women, Personal needs, Personal concerns, Qualitative study, Cultural and religious expectations

## Abstract

**Background:**

Cultural and religious norms and expectations may influence the needs and behavior of single women. This is particularly true in those countries where religion and cultural expectations are salient in everyday life. In this context, the present study investigated the needs and concerns of Iranian never-married women aged 35 and older.

**Methods:**

This qualitative study involved a conventional content analysis. Interviews were done with 23 never-married women aged 36–64 years in Iran.

**Results:**

A total of 773 codes, 22 subcategories, 8 categories, and 3 themes were extracted from the interviews. The 3 themes were: (1) mental-spiritual lack; categories were lack of emotional support, uncertain future, mental rumination, and sexual worries; (2) reform of culture and society; categories were an adverse effect of culture and being overlooked in society; (3) loneliness arising from disability; categories were aging and loneliness and sickness and loneliness.

**Conclusions:**

The results of the analysis indicate that the needs and concerns of never-married women over the age of 35 years in Iran remain unmet. This suggests that policymakers and health planners should take into consideration the growing number of never-married women as a societal reality deserving of attention.

## Background

Maslow’s argument for a hierarchy of needs is based on the idea that the people different needs have ranged from the basic level of physical needs to the highest level of self-actualization [1]. All individuals, whether single or married, have such needs, but such fundamental and individual needs appear to be somewhat overlooked in single people.

According to the International Conference on Population and Development (ICPD), reproductive health refers to a state of complete physical, mental, and social well-being associated with the reproductive system and its processes and functions. Despite an urgent need to improve all aspects of reproductive health, there are unfortunately social barriers and discrimination with respect to access to reproductive health [2].

Studies have shown that single females feel lonely, less attractive, less satisfied with life, and have functional problems with respect to social relationships and self-confidence [3, 4]. Single women also experience status of isolation and stigma [5]. Women in this category are more likely to develop psychological disorders such as depression, aggression, and obsessive-compulsive disorder [6]. They also face problems living independently and communicating with friends [7]. Furthermore, they are likely to experience emotional, psychological, and supportive deprivation, and also to be subject to gender inequality [8]. Sharafoddin (2018) showed that single people are more likely than married people to engage in addiction or suicide [8]. A study by Kajbaf (2008) focused on the psychological consequences of never-married females and males aged 32 to 42 in Iran. Kajbaf found that these people were depressed, socially excluded, and concerned about their finances and personal independence [9]. In contrast, having a partner is claimed to satisfy a person’s need for belonging, and people in committed sexual relationships are happier than single individuals [3]. However, in contradiction with the prior findings, Noorbala (2017) stated that the rate of mental disorders in people who have never been married is less than married people, which is due to the lack of married life difficulties [10].

In contrast to so-called individualistic societies, in collectivistic societies such as those in Africa, South and Central America, Southern Europe, and Asia [11], staying single into old age can be contrary to cultural norms and traditions. These cultures treat marriage in young adulthood as a developmental task [12], and carry the expectation that couple relationships should persist into old age [11, 12]. Against this kind of background, social norms can adversely affect the lives of single women and their reproductive health [13, 14]. For instance, a study on never-married women in Iran found that not being married was considered a social stigma [15]. Every society has values ​​and criteria for evaluating people and placing them in the hierarchy. According to the theory of stigma, single people may not become perfect people and therefore there is a lot of psychosocial discrimination against these people [16]. This issue is especially prominent in Asian countries where marriage is one of the norms of society and not marrying is known as an abnormality and stigma [15]. Qaderzade (2017) mentioned that never-married women face psychological problems, a vague future and feelings of being a burden to others. They usually despise themselves and refuse to see in the community. Thus, isolation and loneliness become more apparent to them [7].

Despite the expectations obtain in collectivistic societies, in recent years the number of single women has increased globally [17]. Statistics also indicate that after the age of 35, the marriage rate for Iranian women decreases [18].

Most studies of single women have focused on young unmarried women, but it is important to consider all age groups and the full range of individual and social characteristics [19]. Furthermore, given that culture (and in particular Islamic culture) permeates all aspects of people’s lives (including values and marriage), the attitudes, values, and concerns of single women in Western countries are different from Muslim women [20]. According to Iranian culture, marriage is highly appreciated, and it is a social norm [21]. Also, having an intact hymen in the time of marriage is a value for the girls and their families. So, in Iran, the only way to have sex, intimate relationship, and become a mother is through marriage [22]. Therefore, never-married women face challenges of their needs such as the sexual desire with religious beliefs, and cultural norms.

Research on the psychosocial health of never-married women in Iran is scarce and consequently little is yet known in any detail about the needs and concerns of this group. Knowing more about these matters would also help inform society’s response and what might be done to address them. To this end, this study tried to answer to the question: “What are the needs and concerns of never-married women over 35 in Iran?“

## Methods

This qualitative study aimed at discovering the needs and concerns of never-married women over 35 years of age in Iran.

### Participants

23 never-married women aged 36–64 years were selected from groups differing in age, education, employment status, living status (living with family or alone), and city of residence through purposive sampling. Inclusion criteria included Iranian nationality and signature of written informed consent (see below). Exclusion criteria were severe mental issues such as substance use disorder, major depressive disorders, bipolar disorders, or schizophrenia (based on self-declarations).

### Data collection

Sampling was purposeful and then snowball method. For sampling, at first, the researcher, with the help of her supervisor, identified and invited the never-married women with inclusion criteria who were working in her school to take part in the study. After that, for access to more participants with more maximum variation, she asked participants to introduce other never-married women between their friends and relatives. On the other hand, the researcher started looking for never-married women among her own relatives and friends, and also she went to religious places that many people, as well as never-married women, go there to pray, too.

At the time of recruitment, participants were informed about the study goals, the confidential situation of interviews, secure data handling, and their voluntary participation. Then informed consent was obtained. The researcher tried to select participants with maximum variation (such as people with elementary education to people with a Ph.D. degree and from academic members to people without having a job, and so on). Data were collected from semi-structured, in-depth, face-to-face, and individual interviews until data saturation. Data saturation means that the interviews were continued until no new data was obtained. In fact, data saturation occurred when a code or a new category did not emerge from data analysis as Strauss (2014) said [23]. The languages of the interviews were Persian. All interviews were recorded. Data were collected from December 2019 to March 2020.

Data triangulation can be reached by collecting data at different times and settings and by using different sampling protocols. In the present study, data triangulation was obtained by using two methods of data sampling (purposeful sampling and snowball sampling).

After drafting the interview questions, if the researcher did not understand some parts of the interview or if further questions emerged, a second interview was arranged. In this study, the number of participants was 23. But five participants were interviewed two times. So, we had 28 interviews with 23 participants. After transcribing some interviews, the researcher confused in some places and did not understand the deep meaning of some participants’ statements, so another interview was necessary. In this case, the researcher contacted the participant, arranged another interview time, and interviewed them again. So, a total of 28 interviews were conducted with 23 participants, and their needs and concerns were examined thoroughly. Each interview lasted about 60–90 min (average 75 min). Interview location was selected according to participants’ preferences and included, health centers, religious institutes, parks, and workplaces. The following questions were posed to gain entry to the interviewee’s world: “In your opinion, what are the needs of never-married women?”; “What is your understanding and experience of such needs?”; “Do such women have preoccupations?” and “How about you?” Analytical questions such as “Can you explain more or give an example?” assisted the researcher during the interviews.

### Data analysis

Data were analyzed using Graneheim and Lundman’s [24] method. For this purpose, it is used conventional content analysis and inductive reasoning method, Using MAXQDA 10 software for data management. This approach helps in avoiding the use of preconceived categories and allows the categories to emerge from the data [25]. The steps are as follows:

1. The recorded interviews were all transcribed, immediately after the interview, and then read through several times to build up a general impression.
2. The text was divided into semantic units that were summarized in the next step and converted into codes.
3. Codes were compared based on their differences and similarities and organized into subcategories based on similarities, in the next step, subcategories were compared with each other, and similar subcategories were combined (based on internal similarities and external differences from other subcategories) that the main categories finally emerge.
4. Finally, the themes were created as latent content in the text.

### Trustworthiness of data

Four Guba and Lincoln [26] criteria [credibility, reliability, generalizability (transferability), and consistency] were used to establish data validity. Initial themes were carefully reviewed by the study team credibility increased through conducting in-depth interviews, member checking, and validation of emerging codes and categories in the subsequent discussion and through comparing and contrasting by two researchers until agreement was reached. The conformability and credibility of the findings were verified through member checking, peer checking, and maximum sampling variation. To create reliability, one expert (the observer not involved in the study) conducted the second reviewing process. About 78 % of the codes and categories were revised to the point at which the study team expressed strong agreement. To reach a conclusive decision, disagreements were modified by discussion. To generalizability, diverse samples of participants were recruited, the researcher proceeded to present an exact description of the study process and the activities performed during the course of the study (the research steps were recorded), and the interviews were analyzed by individuals other than the participants in the study. Finally, to achieve data consistency, the research team reviewed the interviews, codes, subcategories, and categories, again.

## Results

Participants’ socio-demographic characteristics are summarized in Table 1.

**Table 1:** Individual-social characteristics of participants

Number	Age	Education	Job	Living status	Living of city
1	37	MSc	Student	Living with family	Tabriz
2	36	PhD student	Clerk	Living with family	Kermanshah
3	40	MSc	Clerk	Living with family	Karaj
4	45	Elementary school	Clerk	Living with family	Tabriz
5	38	Student	Clerk	Living with family	Sardroud
6	52	BSc	Retired	Living alone	Sardroud
7	40	BSc	Clerk	Living with family	Tabriz
8	38	BSc	Clerk	Living alone	Ilam
9	41	Doctor	Academic member	Living with family	Tabriz
10	42	Doctor	Without hob	Living alone	Ardebil
11	40	PhD student	Academic member	Living with family	Shiraz
12	42	Elementary school	Without job	Living with family	Marand
13	52	High school	Without job	Living with family	Tehran
14	42	MSc	Teacher	Living with family	Mashhad
15	48	Elementary school	Without job	Living with family	Maragheh
16	64	Diploma	Tailor	Living alone	Tehran
17	39	Diploma	Teacher	Living with ffamily	Marand
18	36	PhD student	Student	Living with family	Gorgan
19	40	BSc	Nurse	Living with family	Tabriz
20	38	Diploma	Without job	Living with family	Khoy
21	53	Elementary school	Without job	Living alone	Tehran
22	50	Doctor	Academic member	Living alone	Sari
23	43	Elementary school	Barber	Living with family	Tabriz

A total of 773 codes, 22 subcategories, 8 categories, and 3 themes were extracted from the interviews (Table 2).

**Table 2:** Subcategories, categories, and themes extracted from interviews

Subcategories	Categories	Themes
Need for a companion	Lack of emotional support	Mental-spiritual lack
Need to love and be loved		
Striving to be a mother		
Fear of permanent celibacy	Uncertain future	
Fear of unsuccessful marriage in the future		
Fear of being an imposition on family members		
Psychological insecurity	Mental rumination	
Incessant never-ending despair		
Feeling guilty		
Incessant continuous regret		
Concerns if meeting sexual needs	Sexual worries	
Forced to ignore sexual needs		
Stigma of being single	Adverse effect of culture	Reform of culture and society
Stereotypical thinking of people		
Failure to provide reproductive health services without judgment		
Need to set up partner-finding agencies for singles	Being overlooked in society	
Need for skills to live alone		
Need for financial support		
Fear of aging due to disability	Aging and loneliness	Loneliness arising from disability
Fear of losing fertility		
Fear of emerging diseases	Sickness and loneliness	
Fear of diseases without a supporter		

The most common and powerful theme that was formed from the most codes (465 codes) was labeled “mental-spiritual lack”; the following topics were the basis of the first theme: lack of emotional support, uncertain future, mental rumination, and sexual concerns:

1. Lack of emotional support

This category was formed by combining three sub-categories, (1) Need for a companion, (2) Need to love and be loved, and (3) Striving to be a mother:

One of the unmet needs that the never-married women suffered from was not having a companion. One participant said, *“… When an older person gets married, she actually wants a companion”* (64 years old).

The participants also needed someone to love and to be loved by him. *“I have everything now …, I just want someone to love me”* (38 years old).

From the participants’ point of view, the desire to become a mother is an innate need for women. One participant said, *“Everyone gathers around me calling me ‘Madam or Professor’, but I wish someone would call me Mom instead!”* (41 years old).

2. Uncertain future

This category had three subcategories: (1) Fear of permanent celibacy, (2) Fear of unsuccessful marriage in the future, and (3) Fear of being an imposition on family members.

One participant said, *“I’m always afraid that I’ll grow old and stay single …”* (40 years old).

At the same time, participants were worried about an unsuccessful marriage in the future. *“I’m worried that I can’t be able to cope with married life, and he may not be the person who can comfort me ….“* (36 years old).

Being an imposition on others was another concern of the participants. *“When I see everyone going somewhere with their family, but I have to go with my brother’s family, I feel I’m a burden on their lives”* (38 years old).

3. Mental rumination

This was composed of sub-categories: (1) Psychological insecurity (2) Incessant never-ending despair, (3) Feeling guilty, and (4) Incessant continuous regret formed this category.

One participant, talking about psychological insecurity, said, *“As you grow older, you need mental security; you want to have peace of mind with your husband …”* (39 years old).

Despair was another psychological concern of the participants. *“How can I know whether someone will marry me in the future …”* (43 years old).

Some participants also felt guilty. *“We wronged ourselves by not getting married and hurt ourselves mentally and physically”* (37 years old).

At the same time, the women also had a desire for married life. *“It’s very difficult to see girls younger than you got married, but you got nothing …”* (42 years old).

4. Sexual worries

This category consisted of two sub-categories: (1) Concerns if meeting sexual needs, and (2) Forced to ignore sexual needs.

Concerns about the consequences of meeting sexual needs were seen in the interviews. *“Well, a never-married woman who wants to satisfy her sexual needs in any way will not have a good result and will cause harm …”* (36 years old).

 Some participants had to ignore their sexual needs. *“We do not have a husband to meet this need … So, this feeling is lost in us”* (37 years old).

The second theme in the study (the reform of culture and society) was formed from 211 codes and two categories: 1 adverse effect of culture, and 2. being overlooked in society.

1. Adverse effect of culture

This category had three sub-categories as (1) Stigma of being single, (2) Stereotypical thinking of people, and (3) Failure to provide reproductive health services without judgment.

Stigma was one of the concerns seen in the participants. *“When I say somewhere that I’m not married, people totally change their opinions about me … They think I must have a problem that I’m single”* (42 years old).

Never-married women believed that people’s opinions about them should change. *“When people know a single woman lives in an apartment, they have negative opinions about her … they think their husbands are in danger”* (39 years old).

At the same time, the stigma of receiving reproductive health services and Iranian laws and traditions regarding the hymen was one of the obstacles for participants attending a woman’s clinics for problems with their reproductive system. *“… Once when I went to the hospital for my vaginal infection, the ladies asked me why did you get the infection!?”* (37 years old).

2. Being overlooked in society

This category had three sub-categories: (1) Need to set up partner-finding agencies for singles, (2) Need for skills to live alone, and (3) Need for financial support. Under this heading are the following observations by interviewees:

*“It is necessary to establish institutions for never-married people where they can receive some services … to find someone to marry*“(42 years old).

*“Educational institutions should be established to teach never-married women how to have a single life because no one has taught us this before …”* (52 years old).

Other responses referred to the need to have income and concerns about financial problems. *“My problem today is an economic problem. If I had a salary, I could live very well* …” (64 years old).

The “loneliness arising from disability” theme was formed from the least codes (97 codes) in this study. The theme consisted of two categories: (1) aging, and (2) disease. They felt that aging and disease are difficult given that everyone experiences one if not both, but they are much more difficult for a single person.

3. Aging and loneliness

Two subcategories, (1) Fear of aging due to disability, and (2) Fear of losing fertility formed this category.

One of the things that never-married women mentioned was fear of aging associated with disability. *“When you grow older, your efficiency and power decline. I hope it doesn’t happen to me, but if it does, a bitter future awaits me”* (42 years old).

At the same time, fear of losing fertility with age was another important issue in the lives of the participants. *“One of my worries is that my reproductive age is coming to an end”* (45 years old).

4. Sickness and loneliness

This category also had two subcategories: (1) Fear of emerging diseases, and (2) Fear of diseases without a supporter. Participants stated:

*“… I always say thank God that I am healthy now, but what should I do if I come down with a disease in the future? …”* (58 years old).

*“The only person who takes care of me is my mother, and if one day she dies, who will take care of me when I come down with a disease”* (37 years old).

## Discussion

The key findings of the present qualitative study were that never-married women aged 35 years and older reported particular needs and concerns. Specifically, responses yielded three key themes: mental-spiritual lack, reform of culture and society, and disability-associated loneliness (each of which was comprised of categories and subcategories). The results of the present study add significantly to the current literature in that participants’ views covered a broad variety of neglected emotional, psychological, sexual, and social needs and concerns. The range of responses also provides an illustrative portrait of how their needs and concerns related to culture and of their desire for society and the people within it to view them differently. Results are now discussed in more detail.

Participants in this study wanted to get married to have a companion, to become a mother, to love and be loved. In this respect, the present findings are consistent with the results reported by Greitemeyer (2009); this study showed that single individuals feel lonely and want company in their social lives [27]. Sharp (2007) also found that single women were stressed and worried about being alone, and their greatest concern was not having any children [28]. The family is the most important source of support and intimacy among people [29], but over time, people lose their parents and become lonelier, so they want to have someone who loves them. At the same time, the need to be a mother appears to be innate in every woman [30]. In fact, by becoming a mother, women can have someone to love and be loved by in old age and, because the only way to become a mother in Iran is to get married, the participants wanted to get married.

Study participants also worried that they would remain unmarried for the rest of their lives and would become a burden on their families in their old age. At the same time, they were afraid to enter into unsuitable marriages and thereby to suffer its harm. This finding is consistent with the study by Kajbaf (2008); in this study, never-married women cited one of their concerns as not finding a suitable mate over time and consequently accepting marriage to the wrong person [9]. In Iran, women usually live with their families until marriage. This pattern is also found in other Eastern societies such as Indonesia and Malaysia [31]. When parents pass away, women are sometimes forced to live with their siblings, and even if they have an income, they feel they are a burden. Being a burden on others causes the feeling of self-loathing, insignificance, a diminished sense of belonging, and isolation [32]. Qaderzadeh (2017) showed that never-married women in traditional societies are less likely to live independently and, as they get older, they feel that they are a burden on families [7].

Mental rumination followed by such emotions as psychological insecurity, despair, guilt, and regret from witnessing the lives of married people were frequently seen in the participants’ responses. Having mental security is one of the basic human needs that, if met, can give a person peace of mind [33]. The results of Janicka’s (2015) study showed that married women felt more mentally secure than single women [34]. The reason for their psychological security was probably because they felt valued, and that they had someone to support them in making important decisions. In another study (Soulsby, 2015), married people scored better on mental health than single people [35].

Evidence suggests that in Iran, never-married women over 35 years of age are less likely to get married [36]. Thus, never-married older women gradually lose their hope of finding a husband, and they have negative feelings such as guilt due to not paying attention to God’s orders regarding marriage (The holy Q’uran and religious prophets in Islam strongly recommended marriage), as well as constant regret over their past mistakes. These results are consistent with the findings from Sharp’s (2007) study; In this study, females mentioned that they constantly thought that something was wrong in their lives, and their greatest mistake was that they were getting too old for marriage and having children [28]. In Saili’s (2018) study, never-married women also had feelings such as incompetence, regrets over missed opportunities, and guilt [20].

Fulfilling sexual needs (worrying about the consequences of sexual needs are met and, on the other hand, having to ignore them because they do not have a spouse) was one of the major concerns of participants. Given that the cultural and religious rules of Iran mandate that the only way to satisfy sexual needs is to get married [22], people who do not have a spouse feel they have no choice but to ignore their sexual needs. This finding is consistent with the results of the study by Mroczeka (2013), which was conducted on older single individuals; The participants stated that because they did not have a spouse to meet their sexual needs, they had no choice but to deny them [37]. On the other hand, women who fulfill this need feel guilty because, according to Islam, having sexual relationships outside marriage is unacceptable and a major sin.

Participants also expressed the view that their society’s culture is unfair to single people and should be corrected. In the present study, most participants were concerned about singleness stigma, and they considered this stigma to be a product of society’s stereotypes about single individuals. This stigma caused feelings of low self-esteem, low value, and incompetence in never-married women [15]. These findings confirmed the “Goffman theory”. According to this theory, single people may not be complete and must endure much psychological discrimination and stigmatized [16]. This issue was also evident in the Azmawati (2015) study [38]. In Eastern societies, marriage is considered an important cultural imperative, and celibacy reflects social incompatibility [39]. At the same time, while questioning people’s private lives is quite repugnant in Western societies, in Eastern societies such as Indonesia questions about marital status and children are commonplace in social interactions, and judgments are voiced about the lives of single people [40]. In Iranian society, staying single, particularly in old age, can raise questions in people’s minds and bring compassion to never-married women. Women in this category are more likely to maintain contact with their friends who are also single and refuse to be present too much in public. In contrast, in Western societies, single people have an easier social life and can live more happily [38].

Another problem for single women was the fear of being labeled when in receipt of health services for genital problems. The results of Abedini’s (2014) study on never-married female students showed that these women avoided seeking help from health services for genital problems [41]. As women get older, the incidence of reproductive cancers increases and they need more frequent check-ups [42]. Since in Iran having an intact hymen until marriage is highly valued, this influences the use by never-married women of reproductive health services such as the Pap-smear test is influenced by this culture [43]. The law in Iran treats damage to the hymen, whether accidental or during a routine examination, as an offense [44]. Consequently, even doctors refuse to perform vaginal examinations on never-married women and this can lead to late diagnosis of the disease and impose high costs on them. According to the results of a study, the possibility of damage to the hymen during a routine examination is one of the most important reasons for never-married women not to refer themselves to reproductive health services in Iran [45]. This points to the need for plans to address what is both a legal and a social problem around the genital screening of never-married women. Moreover, such individuals should not be judged when referring to health centers.

Creating agencies that can introduce single people to one another for marriage was a need expressed by several participants in this study. In traditional societies, single women are not supposed to express their feelings or take the initiative when encountering a suitable marriage partner and instead accept that marriage proposal should be made by men. Thus, many of these women, despite knowing of potential appropriate partners, fail to marry. These findings are consistent with Ibrahim’s (2009) study in Malaysia [29].

 Teaching life skills to this group of women was another prominent topic in participants’ statements. The growing number of never-married older women has been a disconcerting development for a traditional society such as Iran [38]. Thus, it is very important to teach singleness life skills to those who do not already have any preparation or training for living a single life. Given the traditional expectation in Iranian society that people will live with their parents and then their spouses and children, there has been no planning to prepare and support single women in living alone. This could be achieved through the provision of classes and training to enable them to live a better and happier single life. However, some studies have shown that single women can actually live better and more happily than married women because they are freer and have better quality contact with friends and family [10, 31]. The reason for this in Muslim countries can be due to the belief in God’s destiny and that God considers the best destiny for them.

In the present study, most women had a job or were looking for a job, but a small number who did not have a job complained about their dependence on their families and considered it one of the duties of the government to give them a job or insure them for old age. Having higher education and a job is one of the most important reasons for not marrying among women, and having a job allows them to live happily, freely, and independently [38, 46]. These findings are consistent with Salama (2018) study that was done on Saudi Arabia [47]. However, some never-married women inadvertently remain single and do not have a job; these women feel anxious and a burden to the family. In fact, not having a job was a problem in the lives of some participants and sometimes causes them to be permanently dependent on their families.

Loneliness arising from aging was another concern of never-married women. Loss of fertility with age was a significant concern in the lives of the participants. Loss of fertility and subsequent anxiety were also apparent among the participants in Saili’s (2018) study [20].

Fear of emerging diseases such as cancer as they aged and, on the other hand, lack of support in times of illness caused concern among participants. Women who have never been married are forced to accept loneliness in old age, and instead of living with husbands and children and receiving support from them, rely on substitutes such as friends [48]. The results of Hanske’s (2016) study on the effect of marriage on breast cancer showed that, at the time of breast cancer diagnosis, significantly more unmarried women than married women were found to be at a more advanced stage of cancer (p > 0.0001), and unmarried women were at greater risk and with a poorer prognosis (AHR 1.35; 95 % CI 1.28–1.43) [49]. The most important problem for such individuals in old age and disease is being alone, which is particularly noticeable with the death of parents and the loss of older supporters.

 Lack of cooperation of some women in the interview was a limitation in this study, though the researcher tried to encourage them to participate by establishing appropriate communication and explaining the confidentiality of information. Also, by extending the study time, the researcher tried to interview more participants to get the necessary information, maximum variation and achieve data saturation.

According to the census in Iran and in the world, the number of never-married women is increasing. In Iran, there is no basic organization to support this group of women. The results of the study revealed that some of these women live in suffering. So, policymakers should pay more attention to this issue. This data can help policymakers and planners to organize some programs to establish more facilities to improve the lives of never-married women.

## Conclusions

This study was the first study that has broken the silence of never-married women in Iran regarding their needs and concerns and showed that they have a variety of neglected needs and concerns in a religious country with specific culture and norms. It showed that although they have some similar needs and concerns (such as emotional needs, sexual needs, the concern of feature, adverse effects of culture, stigma, and feeling of loneliness) with other never-married women all over the world, it presented other aspects of these women’s life concerns such as mental rumination and feeling of guilt about their status that was not mentioned in past studies. As well as these women mentioned that they needed some institutions to meet single men and have more chances for marriage which was new data. Also, they stated that as they don’t educate about single life skills before, they expect from the government some facilities to educate them these kinds of life skills. To meet these needs and address their concerns, the collaborative effort of various organizations, politicians, planners, sociologists, and even families is essential. In the meantime, reforming society’s culture and government support could be very helpful.

## Data Availability

The dataset generated and analyzed during this study contains the interviewee’s quotation which could disclose the identity of participants. Because of the confidentiality of information, especially the challenge examined in this study is critical in many countries, such as ours, the dataset is not publicly available. But upon reasonable request, we can transfer your demand to the corresponding author.

## Abbreviations

AHR: Adjusted hazard ratio
